# Sclerosing lobular hyperplasia of breast: cytomorphologic and histomorphologic features: a case report

**DOI:** 10.1186/1742-6413-3-8

**Published:** 2006-04-03

**Authors:** Payal Kapur, Dinesh Rakheja, Dominick C Cavuoti, Sarah F Johnson-Welch

**Affiliations:** 1 Department of Pathology, University of Texas Southwestern Medical Center, Dallas, TX, USA; 2 Children's Medical Center, Dallas, TX, USA

## Abstract

**Background:**

Mammary sclerosing lobular hyperplasia is an uncommon benign lesion of adolescent and young women. Fine-needle aspiration cytology of mammary sclerosing lobular hyperplasia is said to show characteristic features that include an absence of stromal fragments.

**Case presentation:**

In this article, we describe a case of sclerosing lobular hyperplasia that occurred in the right breast of a 12-year-old girl. Fine-needle aspiration cytology showed some fibroadenoma-like features including the presence of stromal fragments, while branched tubular fragments were not seen. The diagnosis of sclerosing lobular hyperplasia was made on histologic examination that showed preserved acinar architecture with lobular hyperplasia and sclerosis of intralobular and interlobular stroma.

**Conclusion:**

Fine-needle aspiration cytology features of mammary sclerosing lobular hyperplasia are not diagnostic and overlap with those of fibroadenoma; however, a distinction between the two benign entities is of no clinical significance. The definitive diagnosis of sclerosing lobular hyperplasia requires histopathologic evaluation.

## Background

The term "sclerosing lobular hyperplasia" is suggested for a benign proliferative lesion of the breast, characterized by prominent hyperplasia of the lobules and sclerosis of the intralobular and interlobular stroma [1,2]. While the histomorphologic features of this entity have been relatively well-described, there have only been two case reports describing the fine-needle aspiration cytology findings [3,4]. Both these reports emphasized the absence of stromal fragments in the cytologic preparations. Here, we describe a case of mammary sclerosing lobular hyperplasia in a 12-year-old girl in whom the fine-needle aspiration cytology revealed fibroadenoma-like features including the presence of stromal fragments.

## Case presentation

A 12-year-old girl was admitted with three-month history of an enlarging mass in the right breast. There was no nipple discharge and she denied any hormonal or other drug use. Her past medical and surgical history was significant for resection of a right breast lesion six months earlier; that surgery had been performed abroad and the medical records were not available. On examination, the right breast contained a large sub-areolar mass that deviated the nipple laterally. The mass was tender to palpation and the overlying skin was warm. It was not fixed to the skin or underlying chest wall. The contralateral breast was normal. A sonogram and a magnetic resonance imaging scan demonstrated a lobular and relatively well circumscribed mass measuring 15.7 × 13 × 7 cm, which enhanced intensely after contrast administration. The radiologic findings were considered suggestive of a phylloides tumor or a giant fibroadenoma. The patient underwent fine-needle aspiration cytology followed by a right lumpectomy.

## Pathologic findings

**Figure 1:**
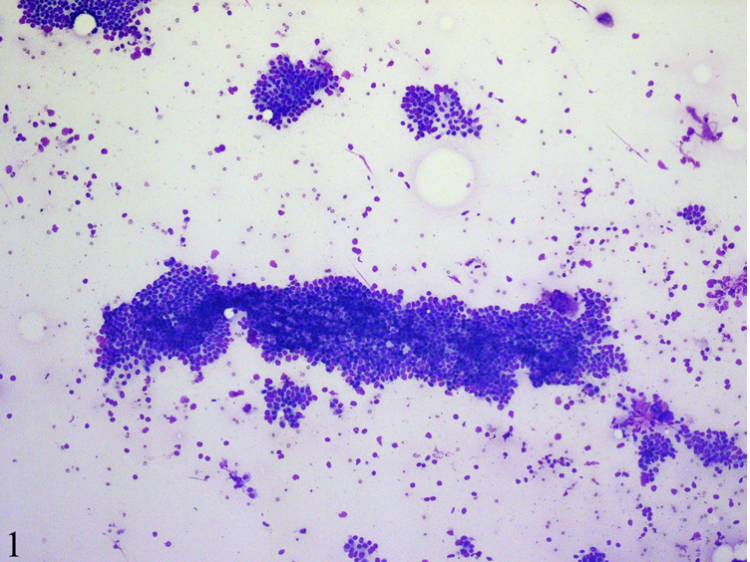
Fine-needle aspiration cytology smears are moderately cellular and comprise of mammary ductal epithelial and myoepithelial cells in clusters and as tubular fragments. The background is relatively clean with a fair number of bare nuclei (DiffQuick, x100).

**Figure 2:**
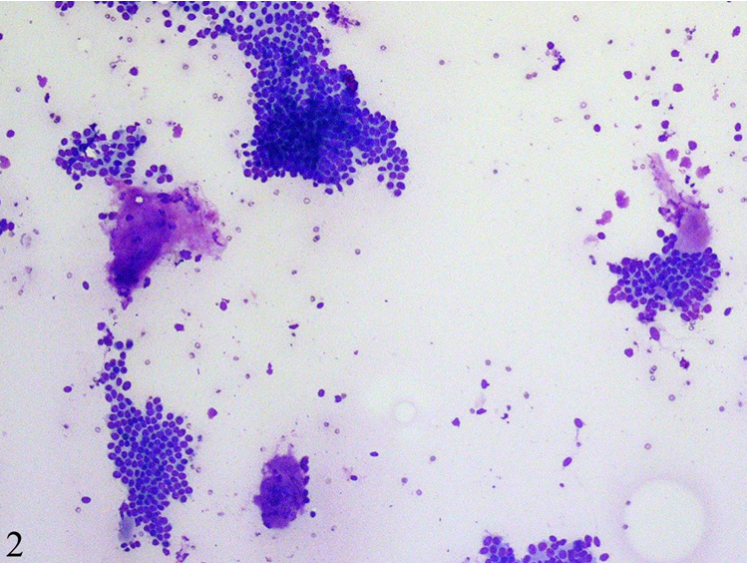
A few scattered fragments of stroma are seen (DiffQuick, x100).

**Figure 3:**
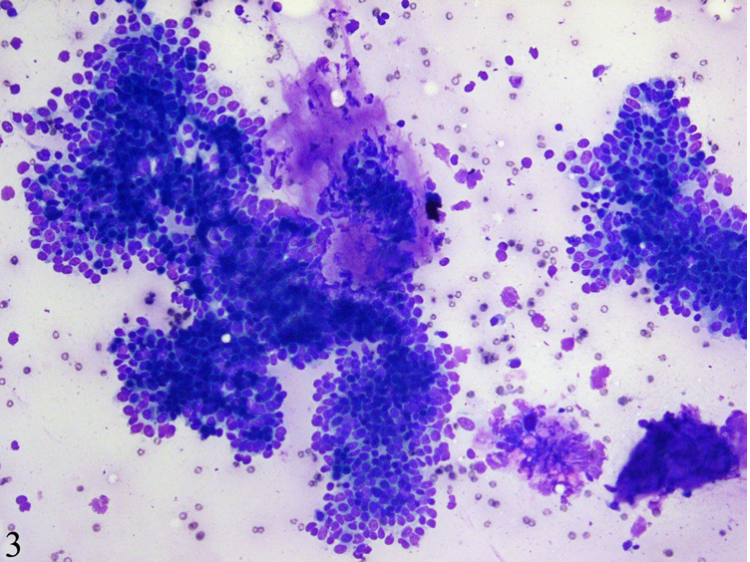
The ductal cells have round to oval nuclei with dispersed chromatin and a moderate amount of basophilic cytoplasm (DiffQuick, x400).

Fine-needle aspiration cytology smears showed a moderately cellular aspirate comprised of bland ductal epithelial cells arranged in small and large clusters and focally long tubular fragments (Figures 1 and 2). "Staghorn-like" branched tubular fragments, typical of fibroadenoma, were conspicuous by their absence. A few scattered fragments of stroma were seen (Figure 2). The background was relatively clean with a fair number of bare nuclei. The ductal epithelial cells had round to oval nuclei with dispersed chromatin and a moderate amount of basophilic cytoplasm (Figure 3). The fine-needle aspiration findings were interpreted as representing a benign mammary epithelial lesion.

**Figure 4:**
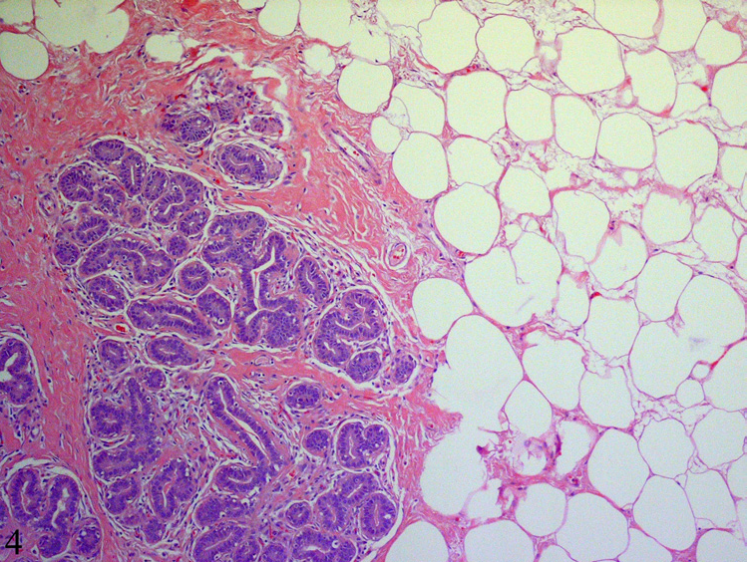
Histologic sections show an unencapsulated but well-circumscribed lesion (Hematoxylin-eosin, x100).

**Figure 5:**
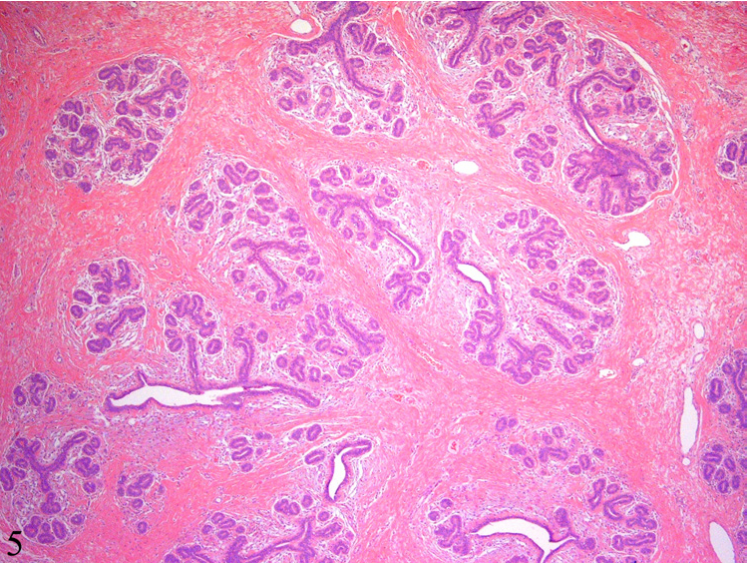
There are multiple large lobules displaying increased numbers of ductules and acini. The intralobular and interlobular stroma consists of dense collagenous fibrosis that is predominantly bland and relatively acellular (Hematoxylin-eosin, x40).

**Figure 6:**
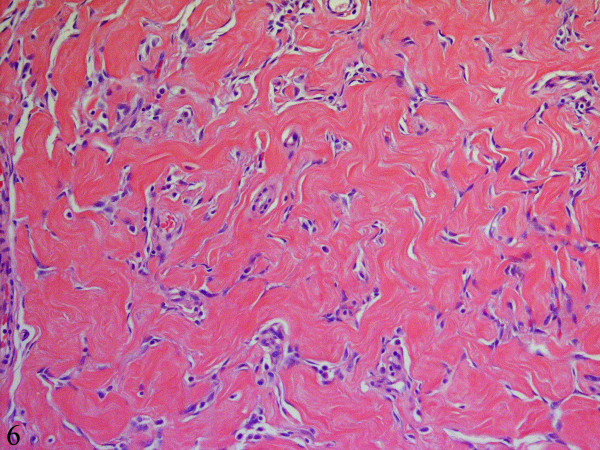
Some foci show dense keloid-like collagenous fibrosis and prominent fibroblastic and myofibroblastic proliferation forming pseudo-vascular slit-like spaces giving the appearance of pseudoangiomatous stromal hyperplasia (Hematoxylin-eosin, x400).

The resected specimen consisted of a 770 g, 20.0 × 14.0 × 4.0 cm nodular and relatively well-circumscribed mass. The cut surface was tan-white, firm, and homogenous with a few scattered slit-like spaces. No areas of necrosis or hemorrhage were seen. Microscopically, the mass was not encapsulated but was well-circumscribed with an abrupt transition between the mass and the surrounding adipose tissue (Figure 4). Low power magnification showed multiple large lobules displaying a proliferation of ductules and acini (Figures 4 and 5). A few ducts were dilated and elongated. There was lobular epithelial hyperplasia with rare mitotic figures and without cellular atypia. The intralobular and interlobular connective tissue consisted of dense collagenous fibrosis that was predominantly bland and relatively acellular (Figure 5). In many areas, however, there was prominent fibroblastic and myofibroblastic proliferation in a background of dense keloid-like collagenous fibrosis. In these areas, the proliferating fibroblasts and myofibroblasts formed pseudo-vascular slit-like spaces giving the appearance of pseudoangiomatous stromal hyperplasia (Figure 6). The stromal cells did not show any atypia and no mitotic figures were identified. A diagnosis of sclerosing lobular hyperplasia was rendered.

## Conclusion

The basic structural unit of the female breast is a lobule, which consists of a varying number of acini. In lobular hyperplasia there is an increase in the number of acini within the lobules. An average of 78.2 acini has been described in hyperplastic lobules, in comparison to 29.6 acini in lobules of normal women aged 20–40 years [1]. Besides lobular hyperplasia, sclerosis of the intralobular and interlobular stroma characterizes the entity of "sclerosing lobular hyperplasia" This entity has also been called fibroadenomatosis, fibroadenomatoid mastopathy, or fibroadenomatoid hyperplasia [1,2].

Sclerosing lobular hyperplasia is an infrequently encountered benign proliferative lesion of the breast. It was first described in 1984 by Kovi *et al*. and is diagnosed in 3–7% of benign breast biopsies [1]. Sclerosing lobular hyperplasia most commonly occurs between the ages of 14 and 46 years [1]. It usually presents as a discrete, palpable, and painless mass in the upper outer quadrant of the breast. Mammography usually reveals a lobular or oval, well-circumscribed mass with echogenic septa that correspond to interlobular sclerosis. Microcalcifications may be seen occasionally, especially in older women. These calcifications may be present in irregular clusters and as granular suspicious lesions. However, distinguishing sclerosing lobular hyperplasia from fibroadenoma on radiologic investigations is difficult in most cases and probably not warranted either, as both lesions are benign and require similar clinical management [5-7].

Fine-needle aspiration cytology findings have been previously reported in two cases of sclerosing lobular hyperplasia [3,4]. Both reports emphasized the presence of ductal epithelial cells in monolayered sheets and of round acinar clusters in a clean background devoid of stromal fragments and with only a few bare nuclei. The cytology findings in our case were similar to those described previously, in the lack of elongated and branched tubular fragments. However, unlike the previously described cases, we saw a few scattered stromal fragments and a fair number of bare nuclei. The sclerosed stroma may make fine-needle aspiration a difficult task and also account for the relative paucity of stromal fragments. However, a complete absence of stromal fragments and rarity of bare nuclei are not consistent features of sclerosing lobular hyperplasia and may not be relied upon to distinguish it from fibroadenoma; a reliable distinction requires histologic examination of the architecture of the two lesions.

Grossly, sclerosing lobular hyperplasia is composed of firm, nodular, tan-white tissue. Histologically, there are enlarged lobules with increased numbers of acini. The intralobular stroma is collagenized with loss of stromal mucopolysaccharides, and there is variable sclerosis of the interlobular stroma [1,2]. Focally, there may be pseudoangiomatous changes in the sclerosed stroma [8]. The lobular acini and ducts have distinct single-layered epithelial and myoepithelial components. Secretory activity is not prominent.

Other palpable breast masses in adolescents include fibroadenoma, juvenile papillomatosis, juvenile hypertrophy, and tubular adenoma. Like sclerosing lobular hyperplasia, fibroadenoma is usually a well-defined, firm to rubbery mass. Histologically, sclerosing lobular hyperplasia is distinguished from fibroadenoma by preservation of the acinar architecture, despite the hyperplastic appearance caused by an increased number of acini per lobule and increased collagenized intralobular and interlobular stroma.

Fibroadenomas, on the other hand, clearly differ from normal acinar architecture with their irregular proliferating elongated and distorted ducts and loose cellular stroma [2]. Sclerosing lobular hyperplasia does appear to be closely related to fibroadenoma. Microscopic fibroadenomas have been described in some sections from sclerosing lobular hyperplasia and sclerosing lobular hyperplasia may occur in the breast tissue surrounding fibroadenomas [1]. The natural course of sclerosing lobular hyperplasia is not known. However, till date no association with or natural progression towards malignancy has been reported. Excision of the lesion is considered adequate therapy. Recurrences have not been documented [6].

In summary, we have described cytologic and histologic features of a case of sclerosing lobular hyperplasia of breast in an adolescent girl. In particular, we wish to emphasize that the presence of stromal fragments in fine-needle aspiration smears from a breast mass does not exclude the diagnosis of sclerosing lobular hyperplasia. Moreover, a "mistaken" diagnosis of fibroadenoma in such cases is of no clinical significance since the two lesions have similar clinical behavior.

## Competing interests

The author(s) declare that they have no competing interests.

## Authors' contributions

PK grossed the surgical pathology specimen, participated in making the diagnosis, and drafted the manuscript.

DR participated in the diagnosis of the case, took pictures for the manuscript, and participated in revising and finalizing the manuscript.

DC performed and read the fine-needle aspiration cytology, and participated in revising the manuscript.

SJW read the surgical pathology sections, and participated in revising and finalizing the manuscript.

All authors have read and approved the final manuscript.
